# From Bench to the Clinic: The Path to Translation of Nanotechnology-Enabled mRNA SARS-CoV-2 Vaccines

**DOI:** 10.1007/s40820-021-00771-8

**Published:** 2022-01-03

**Authors:** Diana O. Lopez-Cantu, Xichi Wang, Hector Carrasco-Magallanes, Samson Afewerki, Xingcai Zhang, Joseph V. Bonventre, Guillermo U. Ruiz-Esparza

**Affiliations:** 1 Division of Engineering in Medicine, Department of Medicine, Brigham and Women’s Hospital, Harvard Medical School, Boston, MA 02115 USA; 2 Division of Health Sciences and Technology, Harvard University - Massachusetts Institute of Technology, Boston, MA 02115 USA; 3 Division of Renal Medicine, Department of Medicine, Brigham and Women’s Hospital, Harvard Medical School, Boston, MA 02115 USA; 4 Tecnologico de Monterrey, School of Engineering and Sciences, 64849 Monterrey, NL Mexico; 5 Department of Cardiovascular Surgery, Union Hospital, Tongji Medical College, Huazhong University of Science and Technology, Wuhan, 430022 People’s Republic of China; 6 Harvard T.H. Chan School of Public Health, Boston, MA 02115 USA; 7 Tecnologico de Monterrey, School of Medicine and Health Sciences, 64849 Monterrey, NL Mexico; 8 John A. Paulson School of Engineering and Applied Sciences, Harvard University, Cambridge, MA 02138 USA; 9 School of Engineering, Massachusetts Institute of Technology, Cambridge, MA 02139 USA

**Keywords:** Nanovaccines, mRNA, Coronavirus, SARS-CoV-2, COVID-19

## Abstract

During the last decades, the use of nanotechnology in medicine has effectively been translated to the design of drug delivery systems, nanostructured tissues, diagnostic platforms, and novel nanomaterials against several human diseases and infectious pathogens. Nanotechnology-enabled vaccines have been positioned as solutions to mitigate the pandemic outbreak caused by the novel pathogen severe acute respiratory syndrome coronavirus 2. To fast-track the development of vaccines, unprecedented industrial and academic collaborations emerged around the world, resulting in the clinical translation of effective vaccines in less than one year. In this article, we provide an overview of the path to translation from the bench to the clinic of nanotechnology-enabled messenger ribonucleic acid vaccines and examine in detail the types of delivery systems used, their mechanisms of action, obtained results during each phase of their clinical development and their regulatory approval process. We also analyze how nanotechnology is impacting global health and economy during the COVID-19 pandemic and beyond.

## Highlights

- Pfizer–BioNTech’s and Moderna’s nanotechnology-enabled mRNA vaccines are the first of its kind to be approved for human use.
- The COVID-19 pandemic has changed our lives and although SARS-CoV-2 has caused irreversible health, social and economic damage, continuous and extensive efforts world-wide were essential to reduce its deleterious effects.

## Graphical abstract

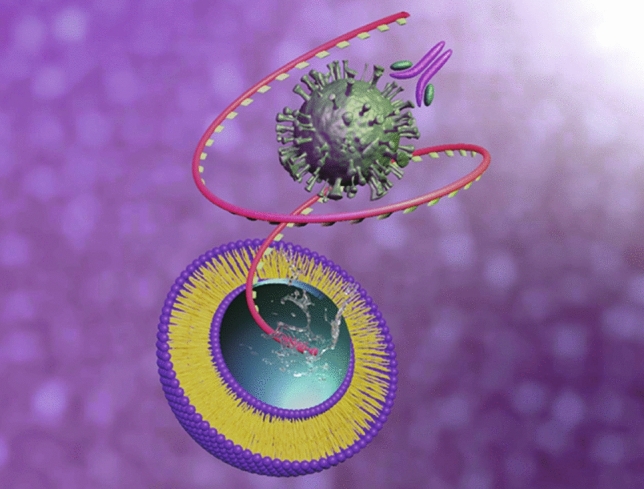

## Introduction

In December 2019, the severe acute respiratory syndrome coronavirus 2 (SARS-CoV-2) was discovered, which then precipitated the emergence of the largest global pandemic since the 1918 Spanish flu which was caused by the Hemagglutinin Type 1 and Neuraminidase Type 1 (H1N1) influenza A virus [1]. The origin of SARS-CoV-2, the virus responsible for coronavirus disease 19 (COVID-19), was traced to bats and pangolins, two mammals that serve as major reservoirs for various types of coronaviruses (CoVs), and many have concluded that human transmission possibly resulted from close interactions with one or both species, nevertheless, more evidence is necessary to confirm this hypothesis [2]. On March 11, 2020, the World Health Organization (WHO) officially categorized this disease as a global pandemic as it spread over the world causing death and economic devastation [3].

Within weeks after the outbreak of SARS-CoV-2 began, a myriad of public health measures that include lockdowns, testing, contact tracing, and hygiene campaigns, were implemented in several countries to control the spread of the virus; nevertheless, millions of people have been infected, and an unprecedented amount of deaths have occurred around the world [4]. Consequently, global financial markets have been very unstable, millions of people have lost their jobs, and a health and economic crisis has emerged. Based on this sanitary emergency, innovative solutions became urgently needed to curtail the spread of SARS-CoV-2.

**Fig. 1:**
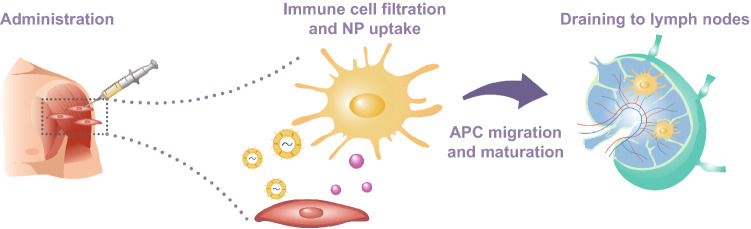
Schematic representation of vaccine administration, nanoparticle uptake by immature APCs, and subsequent migration to lymph nodes through the lymphatic system

In recent years, nanotechnology has been widely used to solve some of the most pressing challenges of modern medicine, as it offers the opportunity to modify and manipulate matter at the nanoscopic scale to generate innovative therapeutic, diagnostic, or theranostic platforms [5]. These technologies include drug delivery nanosystems, nanosensors, nanostructured hydrogels, nanoengineered tissues, and nanovaccines. Nanotechnology has played an important role in the response to the COVID-19 crisis, as various nanoparticle-based vaccines have emerged from several companies around the world. Alliances across industrial (Moderna, Pfizer–BioNTech) and governmental settings allowed the acceleration in the development and clinical translation of nanotechnology-enabled SARS-CoV-2 vaccines. Previously, the development of nano-sized particles formulated to transport antigenic components that induce immune responses has been successfully implemented at the preclinical stage for various applications, such as cancer and some infectious diseases including severe acute respiratory syndrome (SARS) and Middle East respiratory syndrome (MERS) [6]. Nanoparticle-based approaches can achieve targeted delivery of viral proteins or genetic material to antigen-presenting cells (APCs) causing controlled immunogenic responses [7]. Immature APCs uptake nanoparticles (NPs) via phagocytosis or endocytosis and migrate to the closest lymph node through the lymphatic system while undergoing a process of maturation. Once fully matured, APCs complete antigenic presentation on their membrane, CD4^+^ and CD8^+^ T cells are activated, and immunity is produced to target a specific pathogen (Fig. 1) [8].

In this review, we present an overview of the clinical translation of SARS-CoV-2 messenger ribonucleic acid (mRNA) nanotechnology-enabled vaccines, by exploring in detail their mechanism of action and clinical develpopment during the COVID-19 pandemic.

## Coronaviruses

**Fig. 2:**
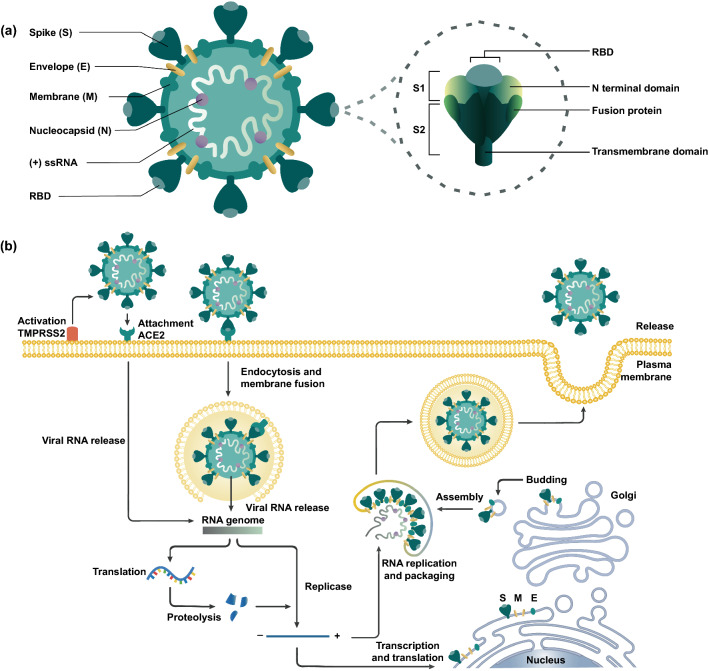
**a** Schematic representation of SARS-CoV-2 and spike glycoprotein main structural features. **b** The viral replication cycle initiates by the activation of the serine protease TMPRSS2 and angiotensin-converting enzyme 2 (ACE2) receptors

CoVs are single-stranded ribonucleic acid (RNA) viruses from the *Coronaviridae* family, with a distinctive crown-like membrane envelope composed of spike glycoproteins localized into their surface [9]. Four genera of CoVs exist: *alphacoronavirus*, *betacoronavirus*, *gammacoronavirus*, and *deltacoronavirus* [10]. To date, seven CoVs are known to affect humans, 229E and NL63 from the *alphacoronavirus* genus, and HKU1, OC43, MERS-CoV, SARS-CoV and SARS-CoV-2 from the *betacoronavirus* genus [11]. Four main structural proteins, essential for the complete assembly of the viral particle are encoded by the coronaviral genome: the spike S protein, the nucleocapsid N protein, the membrane M protein, and the envelope E protein (Fig. 2a) [12]. Each protein has a specific function: the S protein mediates virus adherence to the host cell receptors and subsequent fusion; the N protein binds to the CoV RNA genome, arranges the nucleocapsid and participates in the viral replication cycle; the M protein forms the main structural part of the viral envelope and interacts with all other structural proteins; and the E protein, the smallest integral membrane structural protein incorporated in the viral envelope, is important for the virus production and maturation [13]. The S protein of SARS-CoV-2 consists of two subunits: the S1 subunit contains a receptor-binding domain (RBD) that binds to angiotensin-converting enzyme 2 (ACE2) on the surface of host cells, whereas the S2 subunit mediates fusion between the membranes of the virus and the host cell (Fig. 2a) [14].

SARS-CoV-2, the causative pathogen of COVID-19, has produced a global pandemic due to a highly infectious mechanism based on the co-expression of TMPRSS2 and ACE2 receptors on the cellular membrane of host cells [15] (Fig. 2b). Although ACE2 receptor is expressed on respiratory epithelial human cells, ACE2 is not limited to the lungs, and extrapulmonary spread of SARS-CoV-2 in ACE2-positive tissues has been observed, including the gastrointestinal tract [16–19]. In addition, it has been observed that apical cilia on airway cells and microvilli on type II pneumocytes may be important to facilitate SARS-CoV-2 viral entry [20]. SARS-CoV-2 infection is assisted by TMPRSS2, a cellular serine protease, by two independent mechanisms: cleavage of S glycoprotein to activate host entry, and proteolytic cleavage of ACE2 to promote viral uptake [19, 21, 22]. The priming of the S protein by TMPRSS2 or other proteases is followed by the affinity, binding of the viral S1 protein domain to the ACE2 receptor, and cellular internalization initiated by plasma membrane fusion and acidic-pH-dependent endocytosis [19, 23]. Intracellular replication is then facilitated by RNA-dependent polymerases, and assembly of new viral nucleocapsids from genomic RNA and N proteins occurs in the cytoplasm, whereas new particles are produced by the synergistic action of both the endoplasmic reticulum and the Golgi compartments [14]. Lastly, assembly of the genomic RNA and structural proteins into new viral particles leads to their release via exocytosis [14, 24, 25].

The evolution of SARS-CoV-2 has led to the emergence of multiple variants containing amino acid mutations, some of which have been classified as 'variants of concern' (VOC) that impact virus characteristics, including transmissibility and antigenicity [26]. Reports from several countries on the identification of VOCs (United Kingdom—B.1.1.7 [alpha], South Africa—B.1.351 [Beta], Japan/Brazil—P.1 [Gamma], India—B.1.617.2 [Delta]) and variants of interest (Peru—C.37 [Lambda], Colombia—B.1.621 [Mu], U.S.A.—B.1.427 and B.1.429 [Epsilon]), confirm amino acid substitutions and/or deletions acquired in key antigenic sites, such as the RBD and N-terminal domain (NTD) of the S protein, which facilitate viral cell entry [27–32]. Evidence has shown that some of these mutations (N501Y, particularly) are convergent, arisen independently in different lineages (B.1.351, P.1 [sublineage of B.1.1.28]) [26]. Although no significant evolutionary changes occurred approximately 11 months after the emergence of SARS-CoV-2 in late 2019, multiple mutations were identified since late 2020, and novel lineages are expected to emerge for the duration of the COVID-19 pandemic [26].

Clinical manifestations of COVID-19 may include flu-like symptoms such as cough, fever, and fatigue to more serious clinical consequences including shortness of breath, anosmia, pneumonia, coagulopathy, acute kidney injury, and accelerative inflammation referred to as a cytokine storm [33]. Other manifestations have been reported in the gastrointestinal tract, liver, heart, skin, and central nervous system [34]. High mortality rate and clinical complications of COVID-19 are particularly associated with advanced age, and multiple co-morbidities such as obesity, hypertension, diabetes, and heart disease [35]. As the world faced a fast-evolving and highly contagious threat, innovative approaches were crucial to develop effective vaccines with the aim to suppress the pandemic and decrease mortality.

## Nanotechnology as a Tool to Develop Vaccines

**Fig. 3:**
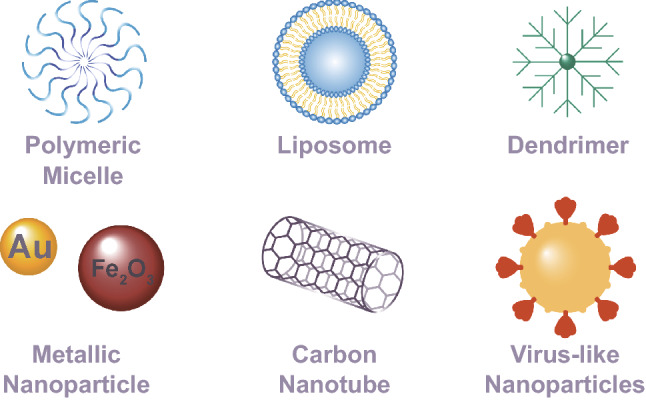
Schematic representation of the structural composition of different types of nanoparticles used for vaccine development

During the last decades, nanotechnology has enabled the development of candidate vaccines for the effective delivery of genetic material and antigenic proteins with high specificity through various administration routes (i.e., oral, intramuscular, intranasal, intradermal, and subcutaneous) [36–41]. Nanovaccination delivery systems have been developed in different forms and can be classified in several categories based on their composition: lipid, polymeric, inorganic, and virus-like nanoparticles (VLNPs) (Fig. 3) [42, 43]. Each class of nanoparticle contains multiple subclasses, with various advantages and disadvantages regarding cargo, delivery, and patient response.

### Lipid-Based Nanoparticles

Lipid-based nanoparticles have been the most common class of nanomedicines approved by the U.S. Food and Drug Administration (FDA) [44]. Lipid-based nanoparticles are excellent platforms for the encapsulation of diverse hydrophobic or hydrophilic therapeutics, including small molecules, proteins, and nucleic acids. Their multiple advantages include formulation and synthesis simplicity, self-assembly, biocompatibility, and high bioavailability [45]. Common fabrication techniques for lipid-based nanocarriers are high-pressure homogenization, high-speed stirring, ultrasonication, emulsion/solvent evaporation, double emulsion, phase inversion, and solvent injection [46].

Lipid-based NPs are divided into several types of systems: liposomal NPs, composed of a lipid bilayer enclosing a hydrophilic core; lipid nanoparticles (LNPs), liposome-like structures with diverse morphologies that usually form an inverse micelle within the core to encapsulate hydrophilic agents; and solid lipid nanoparticles (SLNPs) composed of a lipid monolayer enclosing a solid lipid core [47]. Gene delivery systems often use LNPs to encapsulate nucleic acids in spherical vesicles composed of several materials: ionizable or cationic lipids, helper lipids, and polyethylene glycol (PEG) [48]. Ionizable lipids, which are neutral at physiological pH and positively charged at low pH, are embedded in the micellar structure of LNPs to complex with negatively charged genetic material, aid endosomal escape, and protect against nuclease-mediated degradation [49, 50]. Helper lipids such as distearoylphosphatidylcholine and cholesterol, promote cell membrane binding and provide structural rigidity. Because LNPs can be rapidly taken up by the reticuloendothelial system, PEG is commonly used to decorate the NP surface to increase bioavailability in the human body [51]. Despite these advantages, limitations of lipid-based nanoparticles include low encapsulation efficiency of hydrophobic molecules, poor biodistribution due to a high accumulation in the liver and spleen and, in rare cases, anaphylactic or severe allergy-like reactions as a response to high antibody levels induced by PEG [50, 52].

### Polymeric Nanoparticles

Polymeric NPs can be fabricated with various types of natural materials such as chitosan, chondroitin, alginate, pectin, guar gum, dextran, and xanthan gum [42]. Similarly, synthetic polymeric materials such as polyacrylates, polycaprolactones (PCL), polylactic acid (PLA), poly (lactic-co-glycolic acid) (PLGA), polylactide–polyglycolide copolymers, and charged polymers such as poly(amidoamine) (PAMAM) and poly(ethylenimine) (PEI) have been widely used for nanoparticle fabrication [42, 53]. Techniques for the synthesis of polymeric NPs such as emulsification, nanoprecipitation, ionic gelation, and microfluidics, allow precise control of multiple features including size, shape, charge, surface chemistry, and solubility [54]. Polymeric NPs enable different modalities for delivery: drugs, proteins, or genetic material can be conjugated to the polymer, encapsulated, immobilized into its matrix, or attached to the NP surface [54]. By fine-tunning properties such as composition and surface charge, the loading efficacies, release kinetics, and tissue-specific accumulation of these therapeutics are highly controlled [55].

Polymeric NPs are divided into two main categories, which include nanospheres (matrix systems) and nanocapsules (reservoir systems) [56]. These categories are further divided into micelles, polymersomes, and dendrimers. Polymeric micelles are nanostructures composed of amphiphilic block copolymers that self-assemble into a core shell structure in aqueous solutions [57]. Designing safe and effective micelles that exhibit multifunctional properties by integrating stimuli-sensitive groups and ligands for specific targeting has become especially relevant for the delivery of antibodies and small interfering RNA (siRNA) [57]. Polymersomes, self-assembled vesicles composed of amphiphilic polymers, offer several advantages over traditional liposomes in terms of structural stability; nevertheless, lipid/polymer hybrid vesicles allow a greater control and adaptability of physicochemical properties to any desired functionality or application [58]. Dendrimer NPs, composed of three-dimensional branched synthetic polymers that form interior and exterior layers ideal for molecular conjugation and encapsulation, have been employed in nanovaccination approaches for DNA or RNA delivery [59–61]. Polymeric NPs have been regarded as excellent candidates to deliver molecules due to their biodegradability, water solubility, biocompatibility, stability, and ability to perform targeted delivery. Some disadvantages, however, include risk of particle aggregation and toxicity [54].

### Inorganic Nanoparticles

Inorganic-based platforms composed of metallic (e.g., gold and iron oxide), carbon-based (e.g., nanotubes), or semiconductor NPs (e.g., quantum dots) have been proposed as delivery vehicles for vaccination with promising results [62, 63]. Inorganic NPs exhibit unique size-dependent electrical, magnetic, and optical properties useful for immunological applications through targeting of multiple immune signals, enhanced stability, and delivery of otherwise insoluble cargo [64]. Simple surface modifications allow inorganic NPs to bind to antibodies, drugs, or other ligands and increase their biocompatibility [56]. Some common strategies followed for the fabrication of inorganic NPs include controlled crystallization (solvothermal synthesis or seeded growth), programmed assembly (thermodynamically driven), and templated assembly (coating, casting or breadboard) [65, 66].

Gold nanoparticles (AuNPs) are one of the most well-studied inorganic nanosystems due to their tunable properties and ease of functionalization [67, 68]. Although limited, some inorganic materials such as iron oxide NPs (IONPs) have been FDA-approved for human use, while others are undergoing clinical trials [69, 70]. Besides showing potential as delivery vehicles for antigens and adjuvants, carbon-based nanotubes (CNTs) have shown unique infrared light-responsive properties to induce systemic immune responses [64]. Quantum dots, luminescent nanocrystals with a typical size between 2–10 nm, have been primarily used for in vitro and in vivo imaging applications, nevertheless, they have also shown potential as co-delivery vaccine agents [66, 71]. In general, inorganic NPs are well suited for theranostic applications and offer the advantage of being highly versatile in size, structure, and geometry [54]. Some concerns that arise in the scientific community from these nanomaterials are potential long-term toxicity and limited biodegradability [64].

### Virus-Like Nanoparticles

Immunization strategies have achieved mimicking the conformation of viral structures to create VLNPs or purify viral proteins to synthesize NPs. VLNPs provide several vaccination advantages, including an enhanced uptake through native viral mechanisms and more efficient stimulation of the immune response [72–75]. These nanoparticle systems can serve as vaccination platforms to facilitate the delivery of functionalized or encapsulated adjuvants, antigens, and genetic material that expresses antigenic structures to immunize the organism against pathogens.

VLNP-based vaccines have received attention as they allow the incorporation of ligands, immunomodulators, and targeting moieties into their structure via genetic engineering strategies [76]. VLNPs also offer diverse bioinspired-structures based on human (e.g., Ebola virus, hepatitis B virus, and human immunodeficiency virus) or plant (e.g., Tobacco mosaic virus, Cucumber mosaic virus, Cowpea chlorotic mottle virus) viruses, and modifications include synthetic surface glycans that play a crucial role in modulating protein–receptor interactions, proinflammatory signaling pathways, and cytokine expression [77–82]. Compared to the delivery of live-attenuated or whole-inactivated virus vaccines, VLNPs do not carry the native viral genetic material and thus are safer and non-infectious [83]. As platforms for vaccine development, VLNPs offer the advantage to be highly scalable and adaptable, and some expression systems such as yeast and bacterial cells may greatly reduce their cost during production [76, 84]. Additionally, VLNPs may be designed as multivalent antigen structures, which could provide enhanced cellular uptake and superior immune activation [84, 85]. Despite the potential of VLNPs, some challenges include eliciting the formation of specific protective antibodies, improving the limited duration of immune responses, and effectively mimicking the complex life cycle of some pathogens [85].

### Clinical Development of Vaccines

Several biotechnology companies, hospitals and universities created alliances across industrial and academic settings to rapidly advance basic and clinical research while developing nanovaccination systems during this pandemic (Table 1). For these vaccines to reach the general population, a rigorous clinical development and evaluation process has been followed. According to the WHO, a successful vaccine should reduce disease by at least 50% and show precise information to conclude that vaccine efficacy exceeds 30% (95% CI trial result should exclude lesser efficacies than 30%) [86]. Evaluation from the FDA includes this lower limit of 30% as a criterion for vaccine approval. Part of these efforts resulted in the creation of two highly efficacious vaccines, BNT162b2 by Pfizer–BioNTech and mRNA-1273 by Moderna; both vaccines use nanotechnology as an essential part of their design to deliver mRNA [87].

**Table 1:** Characteristics of SARS-CoV-2 nanotechnology-enabled vaccine candidates.

Candidate vaccine	Developer	Nanoparticle type	Composition	Molecule delivered	Encoded or delivered SARS-CoV-2 antigen	Clinical Trial Number	Number of doses	Vaccine efficacy	Country
BNT162 (a1, b1, b2, c2)	Pfizer–BioNTech	LNP	RNA, lipids ((4-hydroxybutyl)azanediyl)bis(hexane-6,1-diyl)bis(2-hexyldecanoate), 2 [(polyethylene glycol)-2000]-N,N-ditetradecylacetamide, 1,2-Distearoyl-sn-glycero-3- phosphocholine [DSPC], and cholesterol), potassium chloride, monobasic potassium phosphate, sodium chloride, dibasic sodium phosphate dihydrate, and sucrose [177]	a1: uRNA b1: modRNA b2: modRNA c2: saRNA	a1: RBD b1: RBD b2: S protein c2: S protein	Phase I/II NCT04380701, EudraCT 2020–001,038-36 Phase I/II/III NCT04368728	2	82% after first dose against symptomatic COVID-19 [178] b2: 95% [179] 89.5% against B.1.1.7 [134] 75.0% against B.1.351 [134]	Germany, US
mRNA-1273	Moderna/NIAID	LNP	Messenger ribonucleic acid (mRNA), lipids (SM-102, polyethylene glycol [PEG] 2000 dimyristoyl glycerol [DMG], cholesterol, and 1,2-distearoyl-sn-glycero-3-phosphocholine [DSPC]), tromethamine, tromethamine hydrochloride, acetic acid, sodium acetate trihydrate, and sucrose[180]	modRNA	S protein	Phase I NCT04283461 Phase IIA NCT04405076 Phase III NCT04470427	2	82% after first dose against symptomatic COVID-19 [178] 94.1% [178]	US
NVX-CoV2373	Novavax	Protein subunit	Full-length recombinant S protein of SARS-CoV-2 combined with saponin-based Matrix-M™ adjuvant	Protein	S protein	Phase I/II NCT04368988 Phase IIA/B NCT04533399 Phase III EUCTR2020-004,123–16 NCT04583995 NCT04611802	2	Phase IIB 60.1% in HIV-negative; 51.0% against B.1.351 variant and HIV-negative; 49.4% overall [181] Phase III 96.4% against original strain; 86.3% against B.1.1.7/501Y.V1 variant; 89.7% overall [182] Phase III 91% against high-risk populations, 100% against original strain; 90.4% overall [183]	US, Australia
LUNAR-COV19/ARCT-021	Arcturus	LNP	Lipid-enabled and unlocked nucleomonomer agent modified RNA (LUNAR) composed of 50% ionizable amino lipids or MC3, 7% 1,2-distearoyl-sn-glycero-3-phosphocholine, 40% cholesterol, 3% 1,2-dimyristoyl-sn-glycerol and methoxypolyethylene glycol	Self-transcribing and replicating RNA (STARR)	S protein	Phase I/II NCT04480957 Phase IIA NCT04728347	–	–	US, Singapore
LNP-nCoV saRNA	Imperial College London	LNP	Ionizable cationic lipid (proprietary to Acuitas), phosphatidylcholine, cholesterol and PEG-lipid	saRNA	S protein	Phase I ISRCTN17072692	–	–	UK, China
Plant-derived virus-like particle of SARS-CoV-2	Medicago/Glaxo Smith Kline	VLNP	SARS-CoV-2 antigenic proteins	Protein	S protein	Phase I NCT04450004 Phase II/III NCT04636697	–	–	Canada, US
Cucumber mosaic virus-derived VLNP	Saiba	VLNP	Conjugated SARS-CoV-2 antigenic proteins	Protein	S protein	No clinical trial	–	–	Switzerland

*NIAID  National Institute of Allergy and Infectious Diseases, LNP* lipid nanoparticle, *VLNP* virus-like nanoparticle, *PEG* polyethylene glycol, *uRNA* uridine containing mRNA, *modRNA* N^1^-methyl-pseudouridine (m1Ψ) nucleoside-modified mRNA, *saRNA* self-amplifying mRNA, *RBD* receptor-binding domain. S protein, SARS-CoV-2 spike glycoprotein

Once a potential antigen of an infectious pathogen has been identified, the first step involves the development of the mRNA sequence that can express this antigen and its cellular and animal testing (pre-clinical stage) to determine its efficacy [88]. The second step consists of clinical trials, a sequential four-phase process in which the vaccine candidate is tested on humans [89]. During Phase I, small groups of people (hundreds) receive the trial vaccine to evaluate safety and immunogenicity. If satisfactory results are obtained, the vaccine candidate proceeds to Phase II with the objective to expand safety evaluation, identify the optimal dose, and study the efficacy in a larger population (25–1000 or several hundred volunteers) [90]. Phase III trials assess the efficacy of the vaccine in hundreds or thousands of participants, and if other vaccines for the same pathogen exist, a direct comparison is often performed [91].

If favorable results occur in these three phases, an application for registration and approval of a vaccine can be presented to regulatory agencies, such as the FDA in the case of the U.S., which in turn will evaluate the data and make a final decision as to whether it should be approved for clinical use [92]. As all potential short- and long-term adverse events cannot be anticipated until vaccine administration to the general population occurs and time has passed, a Phase IV clinical trial is often done after vaccine approval. This allows for monitoring the safety and efficacy of the vaccine in populations of 100,000 to millions [90]. The probability of success for a vaccine candidate varies by phase and therapeutic area, according to an analysis of Wong et al. that included vaccine candidates from 406,038 trials conducted from January 1, 2000, to October 31, 2015 [93]. The probability of successfully advancing vaccine candidates for infectious diseases to the next clinical phase is 76.8% for Phase I to II, 58.2% from Phase II to III, and 85.4% for Phase III to approval [93]. Approximately one third (33.4%) of the candidates succeed clinical trial phases and reach the public based on this analysis [93].

Generally, vaccine development from conception to approval can take from years to decades. As an example, mumps, rotavirus, and varicella vaccines took four, fifteen, and twenty-eight years to reach the general population, respectively [94]. Nevertheless, because of this global crisis, vaccines against COVID-19 have been developed with unprecedented speed [95]. Each of these stages of pre-clinical and clinical development was speeded up and profuse amounts of investments from the private and public sector were provided to facilitate rapid progress. To accelerate the clinical testing process of a SARS-CoV-2 vaccine, several clinical phases ran in parallel, not sequentially, and in the U.S., Pfizer–BioNTech and Moderna were the first to be granted a fast-track designation by the FDA to expedite clinical studies and approval processes [96, 97]. The accelerated development of vaccines, combined with the novelty of the technologies adopted for their production may result on several concerns, including technical manufacturing problems and ethical matters regarding global access and availability of vaccines [98]. The unprecedented speed in the development of vaccines provides many lessons for the future such as insights on regulations, global access, clinical development, chemistry manufacturing and controls, and post-deployment monitoring [99].

## Differences Between DNA- and mRNA-Based Nanovaccines

The use of nucleic acids is one of the strategies that biotechnology companies and academic institutions have implemented to generate vaccines against SARS-CoV-2. This vaccine type relies on the delivery of genetic information to cells, usually as plasmid deoxyribonucleic acid (pDNA) or mRNA, to encode antigens and induce an immune response in the organism [100, 101]. While mRNA vaccines only need to cross the cellular membrane and reach the cytoplasm of a targeted cell to elicit an effect, pDNA vaccines need an additional step by crossing the nuclear envelope [102]. However, the delivery of unprotected pDNA or mRNA represents a challenge, as enzymatic degradation of the generic material and inefficacy crossing biological barriers such as cellular and nuclear membranes normally occurs [103]. Nanoencapsulation of nucleic acids to produce vaccines has been established as an innovative approach to enable the delivery and protection of genetic material against possible extracellular degradation while preserving its programmed immunologic effect, and several of the nanoparticle-based vaccines against COVID-19 take advantage of this strategy [104, 105].

To fabricate nucleic acid nanovaccines, DNA or mRNA are typically mixed with cationic lipids or polymers to form an electrostatic complex that is subsequently encapsulated into a nanoparticle system [106]. The resulting nanocarrier prevents ribonuclease activity in the genetic construct and facilitates its cellular uptake by APCs via a cell membrane fusion mechanism in the case of most lipid-based systems or through endocytic and phagocytic pathways when polymer-based systems are used [106–109]. One advantage of using nanotechnology-based systems to deliver genetic material is that specialized transfection equipment, such as electroporation or gene guns, is not necessary [110].

**Fig. 4:**
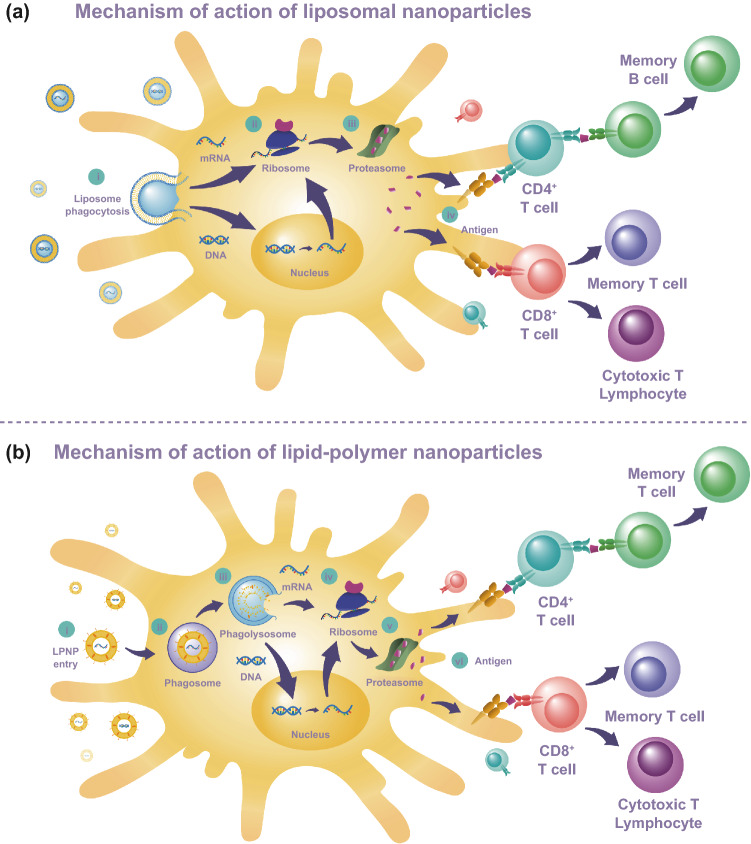
Schematic representation of nucleic acid-based nanovaccination mechanism. **a** Liposomal nanoparticle vaccines (i) The liposomal nanoparticle reaches APC membrane and fuses. (ii) If the cargo is mRNA, it reaches the cytoplasm and is ready for translation. If the cargo is DNA, it must reach the nucleus for transcription into an mRNA molecule. (iii) Subsequently, ribosomes will translate the mRNA molecules into proteins. (iv) Proteasome activity will break the protein down in small antigenic fragments. (v) The antigenic fragments are presented on the APC membrane, and stimulation of the innate immune response is initiated by CD4^+^ and CD8^+^ T cells. **b** Lipid-polymer nanoparticle vaccines (i) lipid polymer nanoparticle (LPNP) reaches APC membrane. (ii) The LPNP is taken up by a phagosome or endosome. (iii) As the phagosome or endosome ages, a phago- or endo-lysosome is formed and later disrupted due to pH changes releasing the genetic material to the cytoplasm. If the cargo is mRNA, it reaches the cytoplasm and is ready for translation. If the cargo is DNA, it must reach the nucleus for transcription into an mRNA molecule. (iv) The ribosomal machinery begins translating the mRNA to produce a protein. (v) Proteasome activity causes the breakdown of the protein in small antigenic fragments. (vi) The antigenic fragments are presented on the APC membrane for stimulation of the innate immune system by CD4^+^ and CD8^+^ T cells

During the internalization process of most lipid-based systems, when the nanoparticle shell integrates into the cell membrane, the genetic material is released directly into the cytoplasm (Fig. 4a) [111]. When hybrid lipid-polymer nanoparticles (LPNPs) with cationic components are taken up into the cell, a phagosome or endosome is formed around the particle and maturation into a phago- or endolysosome will result in its disruption via a pH-dependent proton sponge effect releasing its content to the cytoplasm (Fig. 4b) [112]. In the case of pDNA-based nanovaccines, the genetic material is expected to reach the nucleus where transcription of mRNA molecules will take place, a more complex mechanism when compared to mRNA-based nanovaccines where the genetic cargo just needs to reach the cytoplasm to have an effect [113]. Both approaches result in ribosomal translation, production of antigenic proteins, proteasome activity, and subsequent extracellular presentation of the genetically encoded antigens [114, 115]. After the migration of APCs to local lymph nodes, antigen presentation will trigger cytokine release, induction of cellular responses in CD4^+^ and CD8^+^ T cells, activation of the adaptive immune system and humoral immunity by antibody-producing B cells [115].

Some advantages of using mRNA-based vaccines over their DNA counterparts include their null interaction with the host-cell DNA avoiding possible risks of genomic integration [110]; other benefits when mRNA-based vaccines are compared to viral-based platforms include the absence of anti-vector immunity as it contains an open reading frame encoding the selected antigen and specific regulatory elements which permits its administration multiple times [110].

## The Emergence of mRNA Nanovaccines During the COVID-19 Pandemic

Before the emergency use authorization (EUA) of Pfizer–BioNTech’s and Moderna’s vaccines by the FDA, mRNA-based vaccines have never been FDA-approved in humans for any disease. In the past, however, DNA-based vaccines were already commercially available for veterinary uses such as the prevention of the West Nile Virus in horses and canine melanoma showing no safety concerns [116–118]. In addition, during 2016 and 2017, there were several ongoing human clinical trials evaluating the efficacy of mRNA-based vaccines against cancer and some infectious diseases, [101, 119–121]. Although mRNA technology has shown promising results on in vitro and in vivo models since 1990, there was no substantial investment in developing mRNA therapeutics, mainly because of concerns associated with mRNA instability, high innate immunogenicity, and inefficient in vivo delivery [101, 122]. Novel strategies, including the incorporation of pseudouridine and development of nanoparticle delivery platforms, were crucial for mRNA-based vaccines to emerge as attractive approaches with excellent biocompatibility profiles, facile scalability, and easy manufacturing [101, 123].

Efforts from several companies (Pfizer–BioNTech, Moderna and Arcturus) and academic institutions (Imperial College London) have been made for the development of effective nanotechnology-enabled mRNA vaccines, nevertheless, to date only Pfizer–BioNTech's and Moderna's mRNA vaccines have received emergency approval, and in the next sections of this review article, the preclinical and clinical development of these two SARS-CoV-2 vaccines will be described in detail.

### Pfizer–BioNTech SARS-CoV-2 Vaccine

BioNTech is a biotechnology company that collaborated with Pfizer and Fosun Pharma to test and develop a SARS-CoV-2 vaccine. For this purpose, four nanoparticle-based mRNA vaccine candidates (BNT162a1, BNT162b1, BNT162b2, and BNT162c2) were under investigation. Each candidate possessed a different mRNA format encapsulated in a LNP: two vaccines (BNT162b1 and BNT162b2) contained N^1^-methyl-pseudouridine (m1Ψ) nucleoside-modified mRNA (modRNA); one (BNT162a1) uridine containing mRNA (uRNA); and one (BNT162c2) self-amplifying mRNA (saRNA) [124]. Two of the vaccines had a genetic sequence that expressed the S protein (BNT162b2 and BNT162c2) and the other two expressed the RBD of the spike protein (BNT162a1 and BNT162b1) [125]. The 80-nm sized NPs were composed of ionizable cationic lipids, phosphatidylcholine, cholesterol, and polyethylene glycol [126].

#### Preclinical Studies

In parallel to Phase I/II clinical trials (NCT04380701), antigenicity and immunogenicity of BNT162b1 and BNT162b2 were confirmed in vivo in both murine and primate animal models [127]. First, preclinical studies were performed in BALB/c mice (*n* = 8) by administering 0.2, 1, or 5 μg of the BNT162b1 or BNT162b2, or a buffer as control, using a single-dose regimen. Results showed a high dose-dependent response of either RBD- or S1-specific binding antibodies after the single dose, which increased more steeply for BNT162b2. On day 28, the administration of 5 μg of BNT162b1 was enough to elicit a high RBD-binding response [Geometric mean titer (GMT) = 752,680], similar to that observed when BALB/c mice were immunized with 5 μg of BNT162b2 (GMT = 434,560).

To determine the protective immunity of the nanovaccine, a neutralization assay using vesicular stomatitis virus (VSV)-based SARS-CoV-2 pseudovirus was tested in mouse serum [127]. On day 28 after injection, a steady increase of 50% pseudovirus-neutralization levels were observed after administering 5 μg of either candidate vaccine (GMT = 1,056 for BNT162b1; 296 for BNT162b2). On days 12 and 28 after BNT162b injections, enzyme-linked immunospot assay (ELISpot) showed production of IFN-γ by CD4^+^ and CD8^+^ T cells and IL-2 by CD8^+^ cells in murine splenic T cells. These results were confirmed by intracellular-cytokine-staining flow cytometry analysis after ex vivo restimulation with a full-length S peptide pool.

An additional immunogenic analysis was performed in re-stimulated splenocytes obtained on day 28 from BNT162b-immunized animals with a full-length S peptide pool [127]. After stimulation, IFN-γ and IL-2 secretion was increased in Type 1 helper T-cell (Th1) cells compared to other cytokines, and IL-4, IL-5, or IL-13 in Type 2 helper T-cell (Th2) cells were undetectable. Although similar CD4^+^ and CD8^+^ T cell response patterns were observed for both vaccines, a stronger IFNγ-producing CD8^+^ T cell response was observed in mice inoculated with BNT162b2.

To investigate the principal compartments for T and B cell priming and evaluate systemic effects, the effects of the BNT162b vaccine on proliferation and dynamics of immune cells in draining lymph nodes, blood, and spleen were studied [127]. Twelve days after the administration of 5 μg of either vaccine, an increase of plasma cells, class-switched IgG1^+^ and IgG2a^+^ B cells, and germinal-center B cells in draining lymph nodes were observed, as well as an increase of class-switched IgG1^+^ and germinal-center B cells in spleens of mice, compared to the control. Mice injected with either vaccine also showed an increased level of CD8^+^ and CD4^+^ T cells in the draining lymph nodes, which were notable for T follicular helper (Tfh) cells. Although both vaccines induced higher levels of Tfh cells in the blood and spleen, only BNT162b2 induced an increase of circulating CD8^+^ T cells.

To further test the clinical potential, nonhuman primates were selected in the same study to evaluate the neutralizing response and protective ability of both BNT162b vaccine candidates [127]. Rhesus macaques (*n* = 6, male, 2–4 years old) were administered with two intramuscular inoculations (at a 3-week interval) with 30 or 100 μg of BNT162b1, BNT162b2 or saline control. Results showed detectable levels of RBD-specific binding IgG antibodies by day 14 after one dose and increased levels 7 days after the second dose. On day 28, RBD-binding IgG geometric mean concentrations (GMCs) for BNT162b1 were 20,962 units (U) mL^−1^ and 48,575 U mL^−1^ at 30-μg and 100-μg dose levels, and for BNT162b2 were 23,781 U mL^−1^ and 26,170 U mL^−1^ at 30-μg and 100-μg dose levels, respectively. Compared to the GMCs of RBD-binding IgG of a panel of 38 SARS-CoV-2-convalescent human sera (602 U mL^−1^), the GMCs of inoculated primates were higher after one or two doses.

Neutralizing activity was measured from sera collected 7 or 14 days after the second dose by a SARS-CoV-2 neutralization assay [127]. Results showed that animals administered with 30 μg and 100 μg BNT162b1 had a reciprocal 50% inhibitory dilution (ID_50_) GMT of 768 and 1714, respectively, and those administered with 30 μg and 100 μg BNT162b2 had a ID_50_ GMT of 962 and 1,689. To further investigate the antibody responses for viral inhibition, neutralization GMT of sera collected 21 or 35 days after the second dose from vaccinated animals was  compared to neutralization GMT of human sera from COVID-19 convalescent patients. Results showed that GMT neutralization of macaque sera was substantially higher than that of human samples (GMT = 94).

To determine the protective immunity of the BNT162b2 nanovaccine after one or two doses, ELISpot was employed to evaluate CD4^+^ and CD8^+^ T-cell cytokine specific responses for S protein [127]. Results showed strong IFN-γ but low IL-4 responses after the second immunization, and cytokine staining confirmed CD8^+^ T cells secretion of IFN-γ as well as CD4^+^ T cells secretion of high IFN-γ, IL-2 or TNF levels but low IL-4 levels, indicating a Th1-biased response.

The protective efficacy of both vaccines was further evaluated, as macaques (*n* = 12) previously immunized with either 100 μg BNT162b1 (*n* = 6) or BNT162b2 (*n* = 6), were exposed to a total dose of 1.05 × 10^6^ plaque-forming unit (PFU) of the SARS-CoV-2 USAWA1/2020 strain by intratracheal and intranasal routes forty-one to fifty-five days after the second vaccine dosage was administered [127]. Additionally, control macaques (*n* = 9), previously immunized with saline, received the same viral challenge. Reverse-transcription quantitative polymerase chain reaction (RT-qPCR) analysis was performed in bronchoalveolar-lavage (BAL) fluid, and viral RNA was found in the control group on day 3 (7 of 9) and on day 6 (4 of 8, with one indeterminant result) after challenge; nevertheless, viral RNA was found in BNT162b1-immunized macaques only on day 3 (2 of 6) and not detected in BNT162b2-immunized macaques at any of the time points. In addition, nasal, oropharyngeal and rectal swabs were collected, and results analyzed by RT-qPCR showed viral RNA in the control group on the day after challenge (4 of 9) and in BNT162b2-inoculated macaques (5 of 6), but not from BNT162b1-inoculated macaques. Subsequent nasal swabs showed a decrease of viral RNA detection in control macaques at each sampling time point, a single detection on day 6 from BNT162b1-inoculated macaques, and no detection of BNT162b2-inoculated macaques at any time point. Similar patterns were observed in oropharyngeal and rectal swabs, validating the previous results.

Analysis of the SARS-CoV-2 neutralizing titers on inoculated and control macaques showed values that ranged from 208 to 1185 (BNT162b1), 260 to 1004 (BNT162b2), and undetectable levels (saline) [127]. An increase of SARS-CoV-2 neutralizing titers was observed in control macaques as a response to the viral challenge. Nevertheless, no increase was observed on inoculated macaques with either vaccine, confirming a suppression of SARS-CoV-2 infection. Histological examination was performed in the lungs of the animals, and results showed localized areas of inflammation that were observed among all groups, including the control. This led to the conclusion that the primate animal model was primarily to study SARS-CoV-2 infection rather than COVID-19 disease.

#### Phase I/II Clinical Trials

Using the pegylated lipid nanoparticle system and based on the preclinical results, a combined Phase I/II (NCT04380701), randomized, placebo-controlled, and observer-blinded clinical study among healthy adults was initiated to determine the effective dosage, safety, tolerability, and immunogenicity [128] . The vaccine was administered in a population aged 18 to 55 years old while people aged 65 to 85 years. Three different dose levels of BNT162a1, BNT162b1, and BNT162b2 vaccines following a Prime/Boost (P/B) regimen were under evaluation. In a separate cohort, the BNT162c2 vaccine was administered using a single dose (SD) regimen. The design of the BNT162b1 vaccine is based on the nanoencapsulation of modRNA encoding for RBD of a trimerized SARS-CoV-2 S protein [129]. It has been previously shown that the addition of a trimerization “foldon” derived from bacteriophage T4 fibritin promotes the formation of trimers that allow the presentation of multiple sites for protein–protein interactions [130]. Thus, the genetic material that encodes the RBD antigens was designed with this modification to increase immunogenicity.

The clinical results from three groups of subjects between 18 to 55 years old that were intramuscularly inoculated with the BNT162b1 vaccine at escalating dose levels (10, 30, and 100 µg) and one placebo group were reported [129]. The first group (*n* = 12) received two injections of 10 µg on day 1 and 21; the second group (*n* = 12) received two injections of 30 µg on day 1 and 21; the third group (*n* = 12) received a single injection of 100 µg on day 1; and the fourth group (*n* = 9) received two doses of placebo (control) at day 1 and 21. Seven days after the first and second dose were administered, localized pain at the injection site was the most frequent reaction with mild to moderate severity in all the treatment groups, except for 1 patient who reported severe pain after the first administration of 100 µg. Other experienced symptoms included muscle and joint pain, fatigue, headaches, and chills. Fever was reported after the first and second administrations of BNT162b1, and in the case of the 100-µg group 50% of patients presented side effects; based on this, researchers decided to not administer a second dosage of this dose range. In other groups, only 8.3% had fever which was self-limited after 1 day with no other serious adverse effects reported.

The concentrations of RBD-binding IgG and SARS-CoV-2 neutralizing titers were evaluated before (day 0) and after the first dose was administered [129]. Similarly, titers were assessed again 7 and 14 days after the administration of the second dose. By day 21 after the first dose, GMCs of RBD-binding IgG of the three dosages (10, 30 and 100 µg) were 534–1778 U mL^−1^, compared to 602 U mL^−1^ of the convalescent sera obtained from 38 subjects (18 to 83 years old) 14 days after a COVID-19 diagnosis was confirmed. By comparison, the recipients of the 10 µg presented similar RBD-binding IgG GMC levels to those found in the convalescent sera obtained from COVID-19 patients, whereas the 30 and 100 µg groups had significantly higher titer levels than those measured in the convalescent serum panel GMC.

Seven days after the second dose, the levels increased for 10 and 30 µg dose groups (4813–27,872 U mL^−1^) and highly elevated concentrations persisted until day 35 (5880–16,166 U mL^−1^) [129]. These results represent a ~ 8.0-fold to ~ 50-fold increase in the RBD-binding IgG GMCs compared to convalescent serum panel GMC. RBD binding antibody titers did not increase for the 100 µg dose group beyond 21 days after the first vaccination. Twenty-one days after the first dose of BNT162b1 was administered in all the treatment groups, a modest increase in SARS-CoV-2 neutralizing GMTs was seen. Seven days after the second dose of 10 and 30 µg was administered, 1.8-fold and 2.8-fold higher serum neutralizing GMT levels were detected in comparison to the ones found in the convalescent serum panel from SARS-CoV-2 infected patients. No significant difference in immunogenicity was found between the 30 and 100 µg groups, and the authors concluded that a dose range between 10–30 µg was well tolerated and produced significant neutralizing titers against SARS-CoV-2.

The antibody and T cell responses from the BNT162b1 vaccine were studied in a second non-randomized, open-label Phase I/II (NCT04380701) clinical trial in a population of healthy adults aged 18 to 55 years old [131]. Results indicated that after the administration of two doses of 1 and 50 µg of the vaccine, strong antibody, CD4^+^ and CD8^+^ T cell responses were observed. RBD-binding IgG concentrations were quantified, and superior levels were detected when compared to those found in the COVID-19 convalescent human serum panel. On day 43, neutralizing GMTs from SARS-CoV-2 serum presented an increase of 0.7-fold for the 1 µg dose group and 3.5-fold for the 50 µg dose group in comparison to convalescent human serum. Th1 skewed T cell immune responses with RBD-specific CD8^+^ and CD4^+^ T cell expansion were observed in most subjects and IFN-γ production was detected in both immune cell types. These results indicate that the BNT162b1 vaccine elicits a protective response against SARS-CoV-2.

#### Phase II/III Clinical Trials

As the Phase I/II clinical trial showed promising results, Pfizer–BioNTech decided to subject BNT162b2 into evaluation in a combined Phase II/III (NCT04368728) human clinical trial [125, 132]. BNT162b2, which nanoencapsulates modRNA encoding for SARS-CoV-2 full-length S protein, was initially administered at a 30-µg dose level in a two-dose regimen. The study was estimated to involve 30,000 subjects from 18 to 85 years old, including 120 sites globally.

In December 2020, Phase II/III clinical trials results showed an overall 95% protection efficacy against COVID-19 for the BNT162b2 vaccine [133]. This multinational, randomized, placebo-controlled, observer-blinded clinical trial consisted of a total of 43,548 volunteers (≥ 16 years old) who were randomly assigned at 152 sites worldwide in a 1:1 ratio to receive either the intramuscular vaccine (30 µg) or a placebo, in a two-dose regimen, administered 21 days apart. This study evaluated the efficacy, safety, and immunogenicity of the vaccine for the prevention of COVID-19 illness with onset at least 7 days after the second inoculation in participants who were healthy or had a stable chronic condition (HIV, hepatitis B or C virus infection) and had not previously been infected by SARS-CoV-2 or received an immunosuppressive therapy.

After screening, a total of 43,448 participants received in a 1:1 ratio either BNT162b2 or a placebo, and data were collected on October 9, 2020, from a total of 37,706 participants [133]. The mean age among participants was 52 years, 49% were female, 42% were older than 55 years, 35% were obese and 21% had at least one co-existing medical condition. The racial and ethnic proportions of participants were White (83%), Black or African American (9%) and Hispanic or Latinx (28%).

In the primary analysis for the efficacy of the vaccine, onset cases with no evidence of existing or prior SARS-CoV-2 infection were identified in 162 individuals, 7 days after the second dose in the placebo group, while only 8 onset cases were found in subjects that received the BNT162b2 vaccine, resulting in an efficacy of 95%, in a 95%CI of 90.3 to 97.6 [133]. These results met the prespecified success criteria, and greatly exceeded the minimum FDA criteria (primary efficacy > 30%) for authorization. In the case of participants without evidence of SARS-CoV-2 infection, nine individuals exhibited symptoms of COVID-19 at least 7 days after the second dose of the vaccine, and 169 individuals among placebo recipients (94.6% vaccine efficacy). Severe cases of COVID-19 with onset at any time after the first and second inoculation were identified in 39 individuals of the vaccine group and 82 of the placebo group (52% vaccine efficacy), indicating early protection that starts as soon as 12 days after the first dose.

Safety evaluations considered specific local or systemic reactogenicity and the use of pain medication within 7 days after each inoculation or placebo, and unsolicited adverse events through 1 month and through 6 months, both after the second dose (data through 14 weeks after the second dose is reported) [133]. The most common adverse reaction in participants 16 to 55 years old after the first dose or placebo was injection site pain (83% or 14%), fatigue (47% or 33%), and headache (42% or 34%). In participants over 55 years old, adverse reactions after the first dose or placebo were injection site pain (71% or 9%), fatigue (34% or 23%), and headache (25% or 18%). Most side effects in both vaccine and placebo groups were mild to moderate and were less common in older vaccine groups.

Severe adverse events were identified in 0.6% of vaccine recipients and 0.5% of placebo recipients and both local and systematic reactogenicity resolved shortly after the injection [133]. Although other adverse events included shoulder injury, lymphadenopathy, paroxysmal ventricular arrhythmia, and right leg paresthesia, only a few participants were withdrawn because of serious adverse events. Death cases occurred in six participants during the trial, nevertheless, none of them were considered as consequences from either the vaccine or the placebo. Overall, reactogenicity events were transient and resolved within a couple of days after onset, and serious adverse events were minimal.

As it has been reported, current BNT162b2 efficacy against new SARS-CoV-2 variants could be compromised [135]. For that reason, the implementation of an updated vaccine boost is under clinical review.

### Moderna SARS-CoV-2 Vaccine

Moderna is a biotechnology company based in Cambridge, Massachusetts, which has developed a vaccine candidate (mRNA-1273) by nanoencapsulating a modRNA sequence encoding the S protein of SARS-CoV-2 with 2 proline mutations substituted into residues 986 and 987 (SARS-CoV-2 S-2P) into LNPs [136]. The composition of the nanodelivery system includes ionizable lipids, distearoyl phosphatidylcholine, cholesterol, and polyethylene glycol (molar ratio: 50:10:38.5:1.5) and is fabricated using an ethanol nanoprecipitation method. The nanoparticle size of this vaccine is between 80–100 nm and possesses a mRNA encapsulation efficiency superior to 90%.

#### Preclinical Studies

Prior to human clinical trials, the antigenicity and immunogenicity of this vaccine candidate were confirmed in vitro and in vivo in several murine strains [136]. Preclinical studies were performed in BALB/cJ, C57BL/6J, and B6C3F1/J mice by administering 0.01, 0.1, or 1 μg of the mRNA-1273 nanovaccine using a two-dosage regimen at a 3-week interval. Results showed a dose-dependent response of S-specific binding antibodies in all mice strains after the first (prime) and second (boost) dosages of the vaccine were administered. The administration of 1 μg of mRNA-1273 was enough to elicit a potent neutralizing response, based on the reciprocal IC_50_GMT results, similar to that observed when BALB/cJ mice were immunized with 1 μg of Sigma Adjuvant System (SAS)-adjuvanted S-2P protein. Based on these results, a wider dose range of mRNA-1273 (0.0025–20 μg) was investigated in BALB/cJ mice, and dose-dependent correlations between binding antibodies induced by mRNA-1273 and neutralizing antibodies were observed. To further understand the potential clinical utility of a single-dose vaccination regimen, BALB/cJ mice were immunized with 1 or 10 μg of the nanoencapsulated vaccine mRNA-1273. In 2 to 4 weeks, the group administered with 10 μg developed solid and increasing neutralizing antibody responses as determined by the reciprocal IC_50_ GMT. These data confirmed the efficacy of the SARS-CoV-2 S-2P mRNA nanovaccine to produce neutralizing antibodies with a single dose.

Th1 and Th2 responses were evaluated by comparing the levels of S protein-specific IgG2a/c and IgG1 between the nanovaccine group and a group administered with the SARS-CoV-2 S-2P protein and the TLR4-agonist SAS [136]. The Th1/Th2 response observed was balanced in both groups, as S-binding antibodies, IgG2a and IgG1, were elicited by both immunogens. A single administration of mRNA-1273 resulted in a similar S-specific IgG subclass profile than the two-dose regimen. A different group was administered intramuscularly with the SARS-CoV-2 S protein in combination with 250 μg of alum hydrogel resulting in lower response ratios of IgG2a/IgG1 subclass Th2-biased antibodies. Based on these results, the group concluded that the mRNA-1273 vaccine does not generate Th2-biased responses, which have been related to the vaccine-associated enhanced respiratory disease and observed in children vaccinated with whole-inactivated measles or respiratory syncytial viruses [137, 138].

An immunogenic analysis was performed in splenocytes obtained from mRNA-1273-immunized animals and re-stimulated with different peptide pools (S1 and S2) that correspond to the S protein of the virus [136]. After stimulation, IFN-γ secretion was increased compared to other cytokines such as IL-4, IL-5, or IL-13, and the group stimulated with SARS-CoV-2 S protein combined with alum resulted in a skewed Th2 cytokine secretion. After 7 weeks, intracellular cytokine staining was used to measure the cytokine patterns induced by mRNA-1273 in memory T cells. A dominant Th1 response was found in CD4 + T cells when higher immunogen doses were present; in the case of CD8^+^ T cells, a robust immune reaction to the S1 peptide pool with 1 μg dose of mRNA-1273 was observed. These results indicate that mRNA nanovaccination is able to induce a balanced Th1/Th2 response, compared to the co-administered S protein and alum group where the Th2 response was dominant.

To determine the protective immunity of the nanovaccine, adult BALB/cJ mice were exposed to a mouse-adapted (MA) SARS-CoV-2 that presents localized viral replication in the nasal airways and lungs [136]. Two doses of 1 μg of mRNA-1273 were administered and it was determined that animals were fully protected, as viral replication was undetectable in the lungs after the challenge at 5- and 13-week intervals following a boost. Viral replication was undetectable in nasal turbinates on 6 out of 7 animals. For 0.01 or 0.1 μg dose levels, a dose-dependent efficacy was observed as the lung viral load was reduced ~ 3- and ~ 100-fold, respectively. Animals that received the MA SARS-CoV-2 challenge at 7 weeks after one dose of 1 or 10 μg of the nanovaccine was administered, were completely protected against MA SARS-CoV-2 replication in the lungs. These results confirmed the immunogenicity and efficacy of mRNA-1273 in a murine model and positioned this prototype as a robust candidate for SARS-CoV-2 vaccine.

In a second preclinical study, nonhuman primates were selected to test the neutralizing response and protective ability of this mRNA vaccine [139]. Rhesus macaques (*n* = 24, 12 per sex) were divided into groups of three and administered with two intramuscular inoculations (at a 4-week interval) of 10 or 100 μg of mRNA-1274 in 1 mL of 1X PBS depending on the group. An unvaccinated control group was administered with the same volume of PBS without mRNA.

Results showed a dose-dependent response of S-specific binding IgG antibodies after two vaccinations [139]. Neutralizing activity in animals that received 100 μg of mRNA-1273 had a ID_50_ GMT that was 5 and 18 times greater than the 10 μg dose groups after the first and second vaccination. To further investigate the antibody responses for viral inhibition, serum from vaccinated animals was collected and compared to serum from COVID-19 convalescent human samples. Results analyzed by enzyme-linked immunosorbent assay (ELISA) showed that the binding inhibition of ACE2 to the RBD in serum from 100-μg vaccinated animals was 938 and 348 times higher than that of the control animal group and human convalescent serum, respectively. Additionally, to assess the nanovaccine potential to recognize multiple functional S domains, additive neutralizing activity, post-attachment fusion inhibition, and binding of the NTD of S1 were evaluated. A higher S1 NTD-specific antibody response was elicited by the nanovaccine than the one observed in the human convalescent serum. Finally, the neutralizing activity was evaluated for 10 and 100 μg vaccinated animals using a live SARS-CoV-2 reporter virus, resulting in an ID_50_ GMT 12 and 84 times higher, than the one observed on human convalescent sera.

To determine the protective immunity of the nanovaccine, flow cytometry was employed to evaluate the functional heterogeneity of CD4^+^ and CD8^+^ T-cell cytokine responses specific for S protein [139]. Due to previous observations of robust antibody responses in modRNA vaccines associated with CD4^+^ Tfh cells, interleukin-21 was also measured. Results showed a dose-dependent relationship in increasing Th1, and interleukin-21-producing Tfh cellular responses, and low or undetectable CD8^+^ T-cell and Th2 responses for both dose levels of the vaccine.

The protective efficacy of the vaccine was further evaluated as macaques were exposed to a total dose of 7.6 × 10^5^ PFU of the SARS-CoV-2 USAWA1/2020 strain by intratracheal and intranasal routes four weeks after the second vaccine dosage was administered [139]. Polymerase chain reaction (PCR) analysis was performed in BAL fluid, and subgenomic RNA was found in all the groups at different times; nevertheless, lower levels were observed in the 10 and 100 μg groups compared to the control group.

Post-challenge S- and N-specific IgG antibody levels in BAL fluid were measured to investigate the immune mechanism against viral replication in lungs, and a dose-dependent increase in S-specific IgG antibody levels was detected in vaccinated animals [139]. S-specific IgG responses were higher than IgA responses. No anamnestic response was observed, at 2 weeks post-challenge, humoral S- and N-specific IgG levels were stable in vaccinated animals, in comparison with the increased antibody levels observed in the control group. Histological examination was performed in the lungs of the animals, and the 10 μg group presented mild inflammation without viral RNA. No substantial inflammatory response was found in the 100 μg group as no viral RNA or antigens were detected. These results confirmed that no immunopathologic changes associated with the vaccine were present.

#### Phase I/II Clinical Trials

After obtaining promising preclinical results, a human Phase I (NCT04283461) open-label, dose-escalation clinical trial was initiated to evaluate the security and reactogenicity of mRNA-1273 in 45 healthy adults (18 to 55 years old) [140–142]. The participants were intramuscularly inoculated with 25, 100, and 250 µg of mRNA-1273 (*n* = 15 per group) in a prime-boost regimen 28 days apart. On days 29 and 57 after the first and second inoculation, anti-S-2P antibody GMT was measured by ELISA and results showed that when a higher mRNA-1273 dose was used, an increased antibody response was present. The GMT levels after the first inoculation were 40,227 for the 25 µg group, 109,209 for the 100 µg group and 213,526 for the 250 µg group, and the GMT levels after the second inoculation were 299,751 for the 25 µg group, 782,719 for the 100 µg group, and 1,192,154 for the 250 µg group.

Serum neutralizing activity was measured by both pseudo-typed lentivirus reporter single-round-of-infection neutralization assay (PsVNA) and live wild-type SARS-CoV-2 plaque-reduction neutralization testing (PRNT) assay. PsVNA responses were identified in half of the participants after the first vaccination, and in all the participants after the second vaccination [142]. The geometric mean ID_50_ levels at day 43 were 112.3 for the 25 µg group, 343.8 for the 100 µg group, and 332.2 for the 250 µg group, similar to those found above the median values for human convalescent serum specimens in the distribution. In parallel at day 43, all participants developed a wild-type virus-neutralizing activity that was able to reduce SARS-CoV-2 infectivity by 80% or more (PRNT_80_) (geometric mean of 339.7 for the 25 µg group and 654.3 for the 100 µg group). These responses were higher than the values of three convalescent serum specimens tested. When stimulated by S-specific peptide pools, the 25 and 100 µg groups induced CD4^+^ T cell responses in line with Th1 cytokines (TNF-α, IL-2 and IFN-γ). Th2 cytokine expression (lL-4 and IL-13) was minimal in these 2 groups. Adverse events such as localized pain at the injection site, nausea, arthralgia, fatigue, chills, headache, myalgia, erythema, and induration were observed in a mild and moderate manner after vaccine administration. However, after the first vaccination, the vaccine was considered safe with mild-to-moderate adverse systemic events in 33%, 67%, and 53% of the 25, 100, and 250 µg groups, respectively; after the administration of a second dose, mild-to-moderate adverse systemic events were observed in 54%, 100%, and 100% of the participants in the 25, 100, and 250 µg groups. Severe adverse events were found in three participants (21%) from the high dose (250 µg) group. Fever was present after the second vaccination in 0%, 40%, and 57% of the 25, 100, and 250 µg groups, respectively. It was concluded from these results that the mRNA-1273 vaccine was safe and able to generate immunogenic responses.

On 29 May 2020, a Phase II (NCT04405076) clinical randomized, observer-blinded, placebo-controlled study was conducted to evaluate the safety and immunogenicity of mRNA-1273 in 600 healthy adults (≥ 18–< 55, *n* = 300; and ≥ 55 years old, *n* = 300) [143, 144]. Participants were randomly chosen from either group to receive intramuscular inoculations with either 50 or 100 μg of mRNA-1273 or a placebo in a two-dose regime, 28 days apart. Anti-SARS-CoV-2-spike antibody GMT was measured by ELISA on days 1 and 29 after the first inoculation, and days 43 and 57 after the second inoculation. Results showed that 14 days following the second vaccination (day 43), antibodies were significantly enhanced to maximum GMTs and exceeded those of convalescent sera, remaining elevated through day 57. On day 43, GMT levels (95% CI) were 1733 and 1909 µg mL^−1^ at 50 µg and 100 µg mRNA-1273 in younger adults; and 1827 and 1686 µg mL^−1^ at 50 µg and 100 µg mRNA-1273, in older adults [144].

The most common adverse events were localized pain at injection site, headache, and fatigue following each vaccination in both age cohorts [144]. Local and systemic adverse reactions were mostly mild-to-moderate in severity, at higher frequencies after the second dose, and one serious adverse event that occurred 33 days post-vaccination was concluded unrelated. These observations were consistent with previous reports on Phase I clinical studies. After these results, it was concluded that mRNA-1273 vaccine was safe and able to generate immunogenic responses.

On December 18, 2020, Moderna received investigational new drug approval from the FDA, and the initial placebo-controlled, dose-confirmation Phase I clinical trial was expanded to include older adults (56 to 70 years old/≥ 71 years, *n* = 40) [145]. This study evaluated the safety, reactogenicity, and immunogenicity of a prime-boost regimen of 2 dosages (25 μg, *n* = 10 per age group; or 100 μg, *n* = 10 per age group) of the mRNA-1273 vaccine, administered 28 days apart.

On days 29 and 57 after the first and second inoculation, anti-S-2P antibody GMT was measured by ELISA and results showed that at higher mRNA-1273 doses, antibody responses increased [145]. GMT levels after the second inoculation in the 25 µg group were 323,945 for adults whose age ranged between 56 to 70 years old and 1,128,391 among older adults (≥ 71 years). In the 100 µg group, GMT levels after the second inoculation were far superior to those observed in convalescent sera (GMT = 138,901): 1,183,066 among participants of the 56–70 years old group and 3,638,522 that were 71 years or older.

Serum neutralizing activity was measured by SARS-CoV-2 native spike-pseudotyped lentivirus reporter single-round-of-infection neutralization assay (pseudovirus neutralization assay) against the original virus containing the aspartic acid (D) residue at position 614 [145]. The neutralizing activity of the vaccine against the polymorphic variant 614 glycine (614G) was evaluated with a second pseudovirus neutralization assay on day 43. Additionally, three live-virus neutralization assays were used according to the age and dose subgroup: SARS-CoV-2 nanoluciferase high-throughput neutralization assay (nLuc HTNA) on days 1, 29, and 43 (≥ 56 years, 100-μg dose), a focus reduction neutralization test mNeonGreen (FRNT-mNG) on days 1, 29, and 43 (two age and dose subgroups) and a SARS-CoV-2 PRNT assay (days 1 and 43; two age subgroups, 100-μg dose only).

Pseudovirus neutralization assay responses were identified 7 days after the second dose in participants independently of their age [145]. At day 43, the geometric mean ID_50_ titers to 614D induced by the 100 µg dose level were 402 among adults between ages 56–70 years, similar to 317 observed in adults that were 71 years of age or older. Responses among the 100 µg subgroups were higher than those observed in the 25 µg subgroups and above values in human convalescent serum. On day 43, strong neutralization responses were observed in all participants by nLuc HTNA, similar to those detected by FRNT-mNG. In parallel, on day 43, PRNT_80_ geometric mean levels were 878 among adults between ages 56 and 70 years, and 317 among adults 71 years of age or older. All neutralization assays, nLuc HTNA, FRNT-mNG, and PRNT, correlated well, except results from PRNT and ELISA.

When stimulated by S-specific peptide pools, participants (56–70 years old) that received 25-μg and 100-μg (56 to 70 and ≥ 71 years), induced CD4^+^ T cell responses in line with Th1 cytokines (TNF-α, IL-2 and IFN-γ) [145]. Th2 cytokine expression (lL-4 and IL-13) was minimal in these 2 groups, similar results to those observed in the Phase I trial. Dose-dependent adverse events such as localized pain at the injection site, fatigue, chills, headache, myalgia, were observed in a mild and moderate manner after vaccination. However, after the first inoculation, the vaccine was considered safe with mild adverse systemic events in 30% of the 25 and 100 µg groups (56–70 years), and 50% and 30% of the 25 and 100 µg groups (≥ 71 years), respectively; after the second vaccination, mild-to-moderate adverse systemic severe events were observed in 70% and 88.9% of the 25 and 100 µg groups (56–70 years), and 30% and 70% of the 25 and 100 µg groups (≥ 71 years), respectively. Severe adverse events were found in only two cases: fatigue in one participant (10%) who was 71 years of age or older in the 100-μg dose subgroup, and fever in one participant (10%) between 56 to 70 years old from the 25 µg subgroup after the second vaccination. In addition to Phase II results, it was concluded from this study that the 100-μg dose of mRNA-1273 vaccine was safe, able to generate immunogenic responses and could further proceed for Phase III clinical trials.

#### Phase III Clinical Trials

In late July 2020, the Coronavirus Efficacy (COVE) Phase III clinical trial (NCT04470427) was initiated to evaluate the mRNA-1273 vaccine in preventing SARS infection. Early results from this randomized, stratified, placebo-controlled, observer-blinded Phase III clinical trial, conducted at 99 centers across the USA, were available in February 2021 [146].

The COVE Phase III clinical trial enrolled 30,420 volunteers who were randomly assigned in a 1:1 ratio to receive either the intramuscular vaccine (100 µg) or a placebo, in a two-dose regimen, administrated 28 days apart [146]. This study evaluated the efficacy, safety, and immunogenicity of the vaccine for the prevention of COVID-19 illness with onset at least 14 days after the second inoculation in participants who had not previously been infected by SARS-CoV-2. The mean age among participants was 51.4 years, 47.3% were female, 24.8% were older than 65 years, and 16.7% were under the age of 65 and had high-risk chronic diseases that increased the probability to develop severe COVID-19, such as diabetes, severe obesity, or cardiac disease. The racial and ethnic proportions of participants were White (79.2%), Black or African American (10.2%), and Hispanic or Latinx (20.5%).

The efficacy of the vaccine was evaluated according to the protocol mRNA-1273-P301 based on the most recent clinical study protocol (CSP) Amendment 3 [146]. Immunogenicity analyses included serum binding antibody levels against SARS-CoV-2 as measured by ELISA specific to the SARS-CoV-2 S protein and tests such as VAC58 Spike IgM Antibody, VAC58 Spike IgA Antibody, and VAC65 Spike IgG antibody. Additionally, serum neutralizing antibody titers against SARS-CoV-2 as measured by pseudovirus and/or live virus neutralization assays were performed, including tests such as PsVNT50 PsVNT80, and MN50 (live virus neutralization assay). In the primary analysis for the efficacy of the vaccine, 11 symptomatic COVID-19 participants were identified in the vaccine group and 185 in the placebo group. Secondary efficacy analysis of the vaccine included the identification of severe COVID-19 symptoms among participants. In the placebo group, 30 participants developed severe COVID-19 disease while no cases were observed in the vaccine group. The vaccine showed a 94.1% efficacy, which was similar to secondary analyses, including subjects who had evidence of infection at baseline and analyses of participants 65 years of age or older. Reactogenicity after vaccination was observed to be transient and moderate, which occurred more often in the mRNA-1273 group.

Safety evaluations considered participants’ adverse events (solicited) after the first inoculation, as well as unexpected adverse events (unsolicited), after the second administration [146]. The most common adverse reaction after the two-dose series was injection site pain (84.2%) and systemic adverse events after the first and second dose occurred more often in the vaccine group (54.9% and 79.4%) than in the placebo (42.2% and 36.5%) group after the first dose and the second dose, respectively;; the most common unsolicited events included headache (1.5% vaccine group, 1.2% placebo) and fatigue (1.4% vaccine group, 0.9% placebo). While many of these adverse events were mild (grade 1) or moderate (grade 2), there was a higher occurrence of severe (grade 3) reactions in the vaccine group after the first (2.9%) and second (15.8%) inoculations. Most local adverse events occurred within the first one to two days after injection and generally persisted for a median of one to two days.

The adaptability of mRNA-based vaccines allows the manufacturing of specific nanovaccines against new SARS-CoV-2 variants. For that reason, Moderna will conduct further clinical studies to evaluate mRNA-1273.351 against the variant B.1.351, as well as continue to evaluate a third vaccine boost of mRNA-1273 to determine its efficacy against other new variants that have emerged around the world [147].

## Nanoparticle-Based Vaccines to Deliver Nucleic Acids

As discussed above, Pfizer–BioNTech’s and Moderna’s vaccines were the first vaccines that received emergency authorization by the FDA. For the development of their respective vaccines, Pfizer–BioNTech and Moderna used mRNA that encodes genetic variants of the SARS-CoV-2 spike protein that are more stable and immunogenic than the natural protein. To further increase the efficacy of the formulation, both vaccines use LNPs to encapsulate mRNA molecules to provide them with stability and protection. Advantages offered by this nanosystem include the use of biodegradable lipids, improving safety and tolerability. Other features include the incorporation of multifunctional lipids that act as adjuvants to boost vaccine efficacy [148]. Although immunogenicity of mRNA might represent a safety concern, Pfizer–BioNTech’s and Moderna’s vaccines include chemical modifications to nucleotides (N^1^-methyl-pseudouridine) which reduce mRNA instability and innate immune responses from exogenous mRNA translation [148, 149].

Protein-based vaccines offer different advantages as the protein subunits are readily processed into antigens by APCs, avoiding the need for intracellular transcription. However, exposed antigens are subjected to enzymatic degradation before being recognized by immune cells, which impacts negatively on the vaccine effectiveness and multiple boost doses are usually required. By including adjuvants, however, this intrinsic disadvantage of peptide-based vaccines is countered, allowing the induction of higher immune responses [150].

When comparing mRNA and peptide-based vaccines, one key difference relies on the higher adaptability of mRNA platforms to emergent virus variants or future pandemics. A main concern of genetic vaccines is their safety profile; nevertheless, mRNA vaccines have proven to be not infectious and cannot potentially be integrated into the host genome [151]. Another difference is the storage conditions for each type of vaccine. Protein-based vaccines are stable at temperatures that range 2 to 8 °C and Moderna’s and Pfizer–BioNTech’s mRNA vaccines require temperatures of − 20 °C and − 60 to − 80 °C to remain stable for 6 months, respectively. Although it has been possible to store mRNA vaccines at 2 to 8 °C for 30 days, these conditions still represent a significant challenge for an equitable vaccine distribution to many developing countries [101, 152]. As third doses and seasonal vaccine boosters may become necessary, worldwide efforts continue to ensure health and safety, including the approval of children's vaccination [153–155].

## Health and Economic Impact

Around the world, biotechnology companies and academic institutions accelerated the development of nanotechnology-enabled vaccines with the aim of solving the existent health and economic crisis.

It is imperative to accelerate the manufacturing and distribution of vaccines in a sustainable and equitable manner to rapidly decrease the economic and health impact caused by this pandemic, especially among the most vulnerable and health professionals. A significant difference between mRNA vaccines is the dosage required: Moderna’s vaccine requires 100 μg whereas Pfizer–BioNTech’s vaccine requieres 30 μg. Higher dosages also represent a limitation regarding mass production. In terms of efficacy against the original SARS-CoV-2, Pfizer–BioNTech possess the highest (95%), following by Moderna (94.1%).

In a globalized world, lack of vaccine supply for low-income countries, which are the ones that would likely suffer from limited access, would not only affect public health, but also would take a substantial toll on the global economy. Further consequences would be related to a poor and slow stabilization in the productivity and economy of the world-wide population as the World Bank estimated a 5.3% contraction of the global gross domestic product (GDP) in 2020 [156]. All these effects are aggravated as a significant percentage of patients recovered from COVID-19 will end up with a potential disability due to organic damage, as early evidence has shown that 75.4% of the survivors present abnormalities in lung function [157]. Additionally, 50% of intensive care unit (ICU) patients will experience post-intensive care syndrome (PICS) that includes post-traumatic stress disorder, anxiety, depression, fatigue, insomnia, decreased memory, poor concentration and difficulty talking, and these factors contribute to a loss of economic productivity [158]. Thus, innovation and equitable manufacturing and distribution of effective vaccines will prevent new infections and significantly spur the economic outlook and recovery of societies around the world. Most vaccines developed or under development are based on injectable solutions. In the near future, inhalable vaccines could be created for self-administration without medical assistance, facilitating their distribution and benefiting people worldwide especially those with less developed or limited medical access [159, 160].

Even if vaccines are available, social distancing, use of face covering, and personal hygiene is recommended to control the spread of the pandemic in non-vaccinated populations [161, 162]. Planning security protocols for diverse industrial sectors will also be pivotal to lessen adverse consequences and prevent new outbreaks [163]. In addition, it is crucial to consider the continued emergence of SARS-CoV-2 lineages and their impact on vaccine efficacy. As an example, the lineage B.1.1.7 has been associated with a higher viral load and a reduced neutralization by RBD-specific and NTD-specific neutralizing antibodies (16% and 90%, respectively) [164, 165]. In the case of lineage B.1.351, concurrent mutations have been hypothesized to overcome the polyclonal antibody response due to the significant reduction of neutralizing activity in sera collected from patients recovered from COVID-19 [26, 166]. Besides the effects of mutations on antibody binding and neutralization activity, the biological impact of mutated SARS-CoV-2 variants on T cell reactivity has also been investigated [167]. Tarke et al*.* directly compared SARS-CoV-2-specific CD4^+^ and CD8^+^ T cell responses of COVID-19 convalescent patients previously infected by B.1.1.7, B.1.351, P.1, and CAL.20C (B.1.427/B.1.429) viral lineages, and that were recipients of either mRNA-1273 or BNT162b2 vaccines [168]. Results showed a decrease of 10–22% of the total reactivity, in terms of magnitude and frequency of response, against some VOC combinations. As new variants continue to emerge, information from previous mutations will help to predict altered antigenicity or transmissibility of new viral lineages [169].

Given the urgent need to increase the production and distribution efforts of vaccines against COVID-19, monetary support to scientific and industrial infrastructure is of uppermost importance. These efforts will decrease mortality and disability around the world, and increase the productivity rates across the nations, having permanent long-term economic and health benefits. Monitoring and evaluating long-term adverse effects associated with current mRNA vaccines will also be crucial; however, current clinical evidence supports their short- and long-term biosafety, as severe side effects are rare [170, 171]. To date, two cases of thrombosis with thrombocytopenia syndrome (TTS) have been confirmed following Moderna's mRNA vaccination [171]. Other rare reported side effects after mRNA COVID-19 vaccination are myocarditis and pericarditis. These conditions, however, have been reported as rare events, appearing in 0.1% of vaccinated individuals [172]. As of October 27, 2021, 1,784 reports of myocarditis or pericarditis have been confirmed [171, 173]; further studies are necessary to evaluate whether there is a relationship between these rare adverse events and COVID-19 mRNA vaccination [174]. An additional concern during vaccine administration is the antibody-dependent enhancement (ADE) effect, which has been previously documented for some viral respiratory infections (e.g., syncytial and dengue virus) as it may decrease vaccine success and exacerbate disease [175]. Zhou et al. demonstrated that one group of RBD-specific antibodies found in one convalescent donor induced ADE of entry in Raji cells via an Fcγ receptor-dependent mechanism [176]. Nevertheless, this study concluded that although the ADE effect for coronaviruses may be observed in vitro, a potential pathological relevance during SARS-CoV-2 infection seems unlikely [176].

## Conclusion

SARS-CoV-2 represents a severe health risk to the world’s population, and a rapid and multidisciplinary response has been necessary to overcome this pandemic. At the intersection of engineering and biology, medical nanotechnology, or nanomedicine, as it is more commonly referred to, has proven to be a versatile field that allows researchers to implement novel nanoscopic strategies against new-evolving pathogens. Several biotechnology companies and academic institutions used nanotechnology for the successful design and development of mRNA vaccines against SARS-CoV-2. Based on these results, it is evident that the nanotechnology field has played a pivotal role to prevent further mortality, curtail the pandemic, and aid in the economic recovery of the world.
